# The Tax on Time by Medical Societies

**Published:** 1913-06

**Authors:** 


### The Tax on Time by Medical Societies.

Having within a week resigned membership in one national organization and declined membership in another, it occurred to us to see whether the excuse of lack of time was valid. Here are the actual figures:

| | | |
|---|---|---|
| Local Academy | 33 evenings | |
| County Society | 4 evenings | |
| Special lectures | 3 evenings or parts of afternoons | |
| District Branch | | 1 day |
| State Society | | 3 days |
| Central N. Y. Assn | | 1 day |
| Alumni Assn | | 2 days |
| A. M. A. and coincident societies | | 7 days |
| Totals | 40 evenings | 14 days |

Estimating the evening and afternoon meetings as equivalent to 12 days, the total amounts to 26 days a year. No allowance is made for attendance at other meetings as guest, committee work, special meetings, etc. Several of the organizations mentioned above have, at times, held an additional session. The final sessions of the state and national organizations are not counted, nor is time in traveling. No account is taken of non-medical scientific societies, and the various special scientific medical societies, sociologic organizations and the like, with which so many physicians are connected. While practically all of the organizations mentioned involve business and recreation, the time is estimated on the basis of working days devoted to technical study. In this respect, medical conventions bear little resemblance to ordinary professional and business conventions.

With allowance for Sundays, therefore, the physician who attends regularly meetings of only those organizations which may be considered a duty and not a choice, spends the equivalent of a month of diligent college work—a month without half or whole holidays and, therefore, equivalent to more than an eighth of a college year.

